# Mutations in the postsynaptic density signaling hub TNIK disrupt PSD signaling in human models of neurodevelopmental disorders

**DOI:** 10.3389/fnmol.2024.1359154

**Published:** 2024-04-04

**Authors:** Jianzhi Jiang, Brent Wilkinson, Ilse Flores, Nicolas Hartel, Simeon R. Mihaylov, Veronica A. Clementel, Helen R. Flynn, Fowsan S. Alkuraya, Sila Ultanir, Nicholas A. Graham, Marcelo P. Coba

**Affiliations:** ^1^Zilkha Neurogenetic Institute, Keck School of Medicine, University of Southern California, Los Angeles, CA, United States; ^2^Mork Family Department of Chemical Engineering and Materials Science, University of Southern California, Los Angeles, CA, United States; ^3^Kinases and Brain Development Laboratory, The Francis Crick Institute, London, United Kingdom; ^4^Proteomics Science Technology Platform, The Francis Crick Institute, London, United Kingdom; ^5^Department of Translational Genomics, Center for Genomic Medicine, King Faisal Specialist Hospital and Research Center, Riyadh, Saudi Arabia; ^6^Department of Psychiatry and Behavioral Sciences, Keck School of Medicine, University of Southern California, Los Angeles, CA, United States; ^7^Department of Physiology and Neuroscience, Keck School of Medicine, University of Southern California, Los Angeles, CA, United States

**Keywords:** PSD signaling, induced pluripotent stem cell (iPSC), glutamatergic neurons, proteomics, neurodevelopment, phosphoproteomics

## Abstract

A large number of synaptic proteins have been recurrently associated with complex brain disorders. One of these proteins, the Traf and Nck interacting kinase (TNIK), is a postsynaptic density (PSD) signaling hub, with many variants reported in neurodevelopmental disorder (NDD) and psychiatric disease. While rodent models of TNIK dysfunction have abnormal spontaneous synaptic activity and cognitive impairment, the role of mutations found in patients with TNIK protein deficiency and TNIK protein kinase activity during early stages of neuronal and synapse development has not been characterized. Here, using hiPSC-derived excitatory neurons, we show that TNIK mutations dysregulate neuronal activity in human immature synapses. Moreover, the lack of TNIK protein kinase activity impairs MAPK signaling and protein phosphorylation in structural components of the PSD. We show that the TNIK interactome is enriched in NDD risk factors and TNIK lack of function disrupts signaling networks and protein interactors associated with NDD that only partially overlap to mature mouse synapses, suggesting a differential role of TNIK in immature synapsis in NDD.

## Introduction

Synaptic proteins, specifically those linked in large protein interaction networks (PINs) at the postsynaptic site of glutamatergic neurons (O’Roak et al., 2012; Ting et al., 2012; Li et al., 2016, 2017; Wilkinson and Coba, 2020), have been associated with a number of neurodevelopmental disorders (NDDs; Endele et al., 2010; Peca and Feng, 2012; Genovese et al., 2016; Kilinc et al., 2018; Satterstrom et al., 2020; Trubetskoy et al., 2022). While there is mounting evidence for their function in mature rodent synapses, their role in immature human neurons is not well characterized. The Traf and Nck interacting kinase (TNIK) is a typical example of a postsynaptic density (PSD) signaling hub, associated with NDD (Wang et al., 2010; Coba et al., 2012; Burette et al., 2015; Li et al., 2017). TNIK belongs to the germinal center kinase (GCK), member of the Ste20 family of kinases, and is composed of a ser/thr kinase domain and a citron homology (CNH) domain of unknown function (Fu et al., 1999). The absence of TNIK protein is causative of intellectual developmental disorder (IDD; Anazi et al., 2016), and a number of TNIK variants have been associated with psychiatric and NDD (Potkin et al., 2009; Iossifov et al., 2014; Genovese et al., 2016). Knockdown of TNIK has been shown to decrease dendritic complexity (Hussain et al., 2010; Kawabe et al., 2010) resulting in abnormal spontaneous activity in cultured rodent neurons (MacLaren et al., 2011). In addition, TNIK knockout mice have cognitive impairment and reduced adult neurogenesis (Coba et al., 2012). As a signaling hub, the TNIK interactome shows that TNIK is associated with the core scaffold machinery of the PSD in adult mouse synapses. Patients with the homozygous TNIK pArg180* (R180X) mutation show no TNIK protein and have been diagnosed with recessive IDD, delayed speech, and attention deficit hyperactive disorder (ADHD). However, the role of mutations found in patients with TNIK protein deficiency and the lack of TNIK protein kinase activity during early stages of neuronal and synapse development has not been characterized. Moreover, it is not known whether TNIK associates and dysregulates the same PINs and pathways as observed in mature rodent synapses.

iPSC-derived neurons have been used extensively to model the effect of mutations observed in neurodevelopmental and psychiatric disorders. They share similar expression profiles to fetal cerebral cortex neurons and electrophysiology properties resembling immature neurons (Fink and Levine, 2018; Carter et al., 2020; Lähnemann et al., 2020), making them a suitable tool to address the role of synaptic proteins at early stages of human synapse development. Here, we show that the TNIK pArg180* (R180X) patient-derived mutation and the lack of TNIK protein kinase activity impair neuronal function by disrupting signaling networks and TNIK protein interactors associated with NDD in hiPSC-derived neurons. TNIK protein interactors in iPSC-derived neurons only partially overlap to mature rodent TNIK PSD PINs, suggesting that TNIK regulates a different subset of NDD risk factors in immature synapses. Finally, we show that the lack of TNIK protein kinase activity is enough to dysregulate core cytoskeletal and scaffold components of the PSD signaling machinery and MAPK signaling in immature glutamatergic synapses.

## Results

### TNIK truncation mutation p.Arg180* (R180X) in NDD

**Figure 1:**
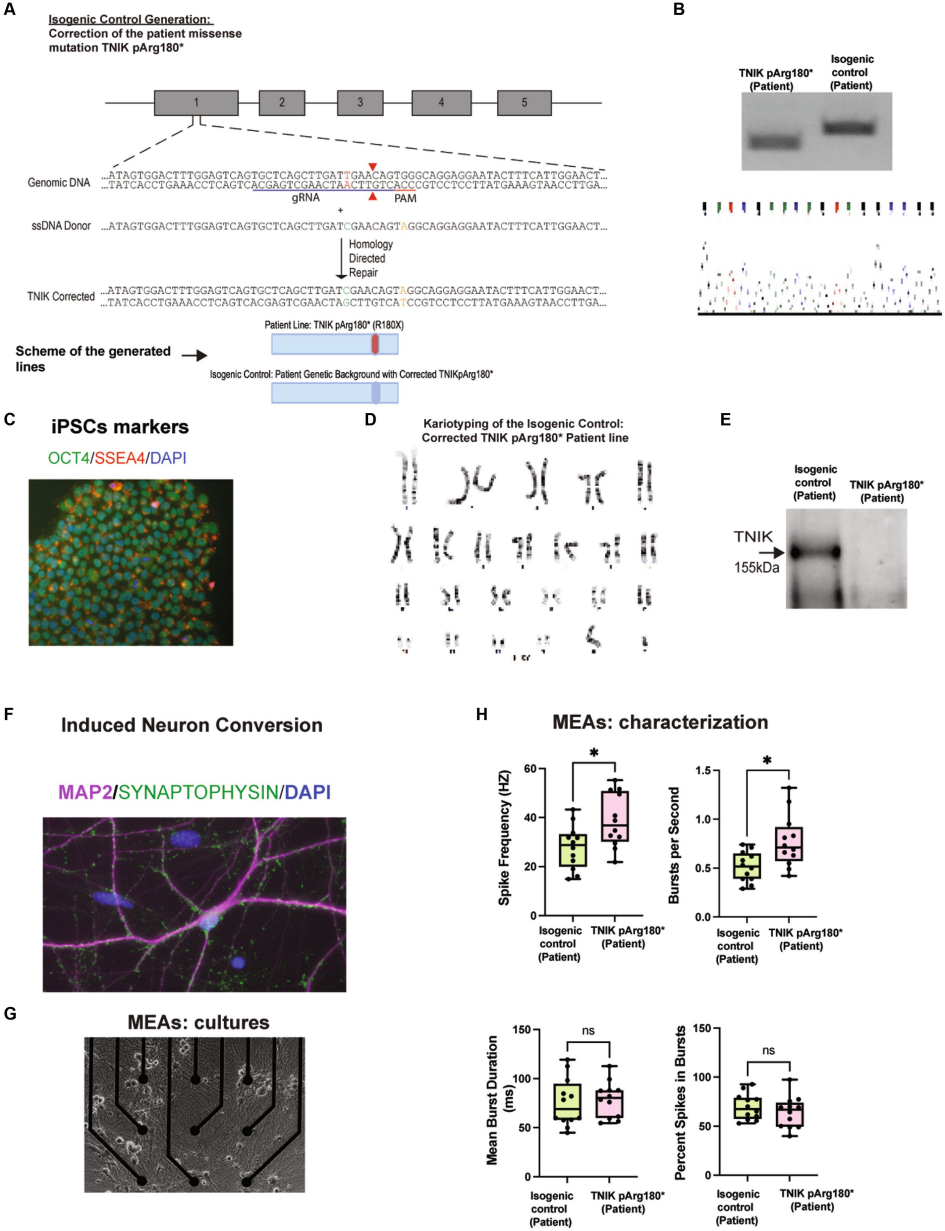
Generation and characterization of TNIK p.Arg180*(Patient) and the isogenic control: Corrected TNIK pArg180* (Patient) cell lines. **(A)** Diagram illustrating the generation of the novel isogenic control TNIK cell line derived from a patient carrying a homozygous p.Arg180* (R180X) truncation mutation. It was performed by CRSPR/Cas9 genome engineering, including guide RNA, ssDNA donor, and HDR (Top Panel). Schematic representation of the cell lines generated (Bottom Panel). **(B)** Agarose gels showing polymerase chain reaction followed by restriction enzyme digest screening for TNIK p.Arg180* (Patient) cell line and the isogenic control (Top panel). Sanger sequencing confirmation for the isogenic control cell line: Corrected patient missense mutation TNIKpArg180* (bottom panel). **(C)** Expression of pluripotent stem cell markers OCT4 and SSE4 in TNIK p.Arg180* (Patient) iPSCs. **(D)** Karyotyping of the isogenic control (corrected TNIK pArg180* Patient line) shows no gross abnormalities. **(E)** Western blot (WB) showing the homozygous TNIK p.Arg180* (Patient) truncation mutation generated a cell line with no detectable TNIK protein levels compared with its isogenic control. **(F)** Induced neuronal conversion of the TNIK p.Arg180* (Patient), and its correspondent isogenic control using Neurogenin-2 (NGN2) induction (iN). **(G)** Multi-electrode array (MEA) cultures of iPSC-derived neurons. **(H)** Recording of the electrical activity of iPSC-derived neurons using multi-electrode arrays (MEAs): TNIK cell line containing the patient-derived mutation TNIK pArg180* (Patient) shows increased spike frequency and number of bursts per second, compared with its isogenic control. Each data point represents a recording well. Detection thresholds were set to a fixed level of −25 μV. * *p* < 0.001.

To start to address the role of TNIK in immature neurons, we generated a novel TNIK cell line derived from a patient carrying a homozygous p.Arg180* (R180X) truncation mutation [TNIK pArg180*(Patient); Anazi et al., 2016], along with their correspondent isogenic control [isogenic control (patient)]. We used episomal expression of Yamanaka factors in patient-derived PBMCs (Okita et al., 2013), together with an HDR template containing the WT TNIK sequence (Figures 1A–D). We then performed a direct neuronal conversion of the TNIK p.Arg180* (Patient) and its correspondent isogenic control using Neurogenin-2 (NGN2) induction (NGN2 induced neurons-NGN2-iN; Zhang et al., 2013; Figure 1F). As previously reported in patients (Anazi et al., 2016), the homozygous TNIK p.Arg180*(Patient) truncation mutation generated a cell line with no detectable TNIK protein as observed by Western blot (WB; Figure 1E) and mass spectrometry assays (Supplementary Table 1). We previously reported an increase in baseline synaptic transmission in adult TNIK^−/−^ mice (Coba et al., 2012). To determine whether TNIK protein is necessary for synaptic function in immature glutamatergic neurons, we recorded the electrical activity of iPSC-derived neurons using multi-electrode arrays (MEAs; Figure 1G). We observed that the TNIK p.Arg180* (Patient) cell line showed an increase in the spike frequency and in the number of spikes per second with no change in the duration of bursts or the number of spikes within bursts (Figure 1H). This suggests that TNIK protein is necessary for normal neuronal function in immature excitatory neurons.

**Figure 2:**
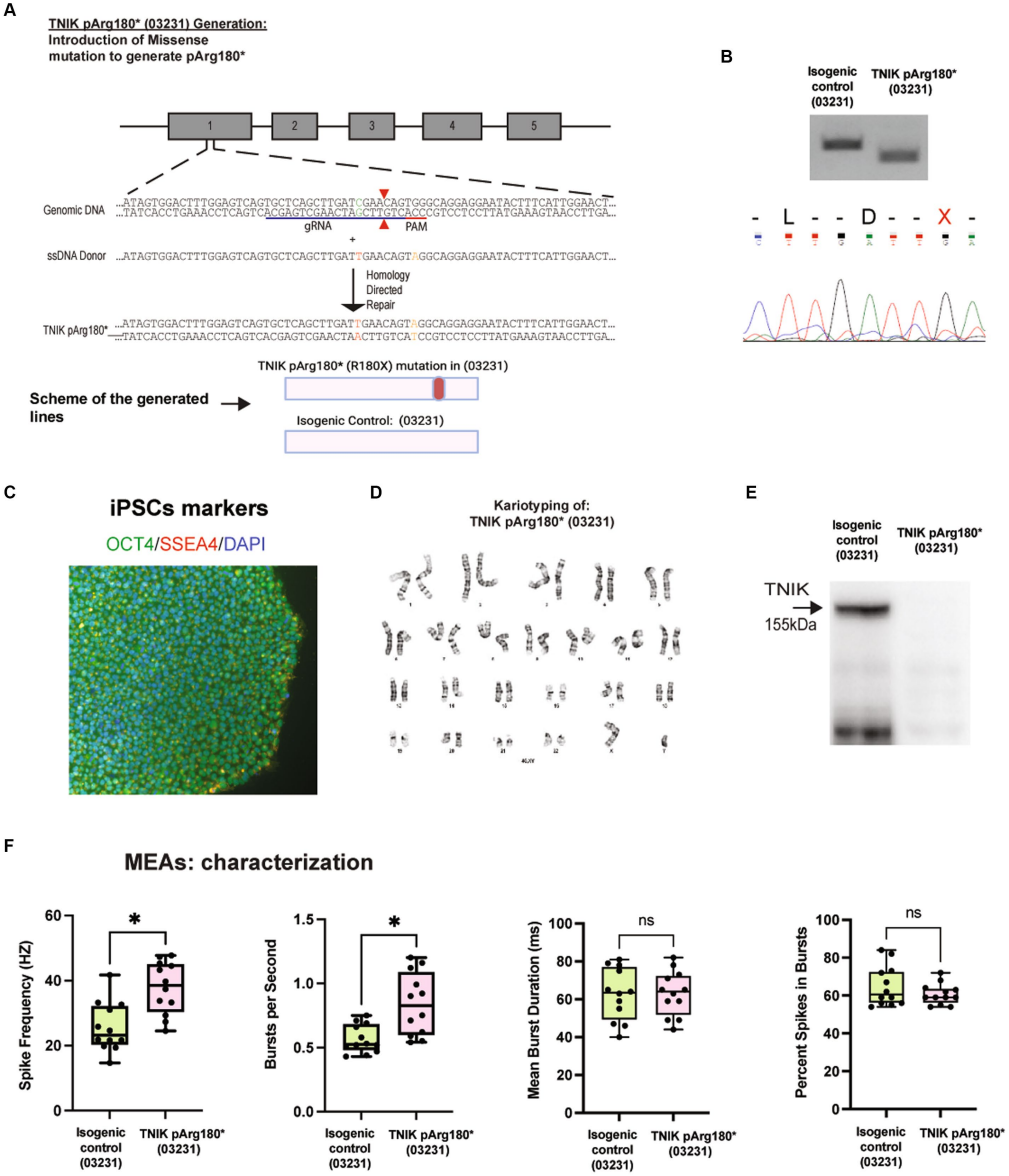
Generation and characterization of the TNIK p.Arg180* (03231). **(A)** Diagram illustrating the generation of TNIK p.Arg180* cell line by CRISPR/Cas9 genome engineering in WT iPSCs 03231, including guide RNA, ssDNA donor, and HDR. (Top Panel). Schematic representation of the cell lines generated (Bottom Panel). **(B)** Agarose gels showing polymerase chain reaction followed by restriction enzyme digest screening for TNIK p.Arg180* (03231) cell line and the isogenic control (Top panel). Sanger sequencing confirmation of cell lines homozygous for TNIK p.Arg180* (03231; Bottom panel). **(C)** Expression of pluripotent stem cell markers OCT4 and SSE4 in the TNIK p.Arg180* (03231) iPSCs. **(D)** Karyotyping of TNIK p.Arg180* (03231) cell line shows no gross abnormalities. **(E)** Western blot (WB) showing the homozygous TNIK p.Arg180* (03231) truncation mutation generated a cell line with no detectable TNIK protein levels compared with its isogenic control. **(F)** Recording of the electrical activity of iPSC-derived neurons using multi-electrode arrays (MEAs): TNIK cell line containing the patient-derived mutation in the WT03231 background [TNIK pArg180* (03231)] shows increased spike frequency and number of bursts per second, compared with its isogenic control. Each data point represents a recording well. Detection thresholds were set to a fixed level of −25 μV. *p < 0.001.

To confirm that the observed phenotype was independent of the patient’s genetic background, we generated a TNIK p.Arg180* (R180X) using the previously reported WT iPSC line 03231 (Wilkinson et al., 2019), via CRISPR/Cas9 genome engineering (TNIKpArg180*; 03231; Figures 2A–D). Sanger sequencing and PCR followed by restriction enzyme digest and screening confirmed the generation of the homozygous TNIK p.Arg180* (03231; Figure 2B) Immunoblot of TNIK in iPSC-derived neurons confirmed that the TNIK pArg180*(03231) cell line does not express TNIK protein (Figure 2E) as reported previously in patients (Anazi et al., 2016) and observed in the patient-derived iPSC line, TNIKpArg180*(Patient; Figure 1E). We then confirmed that the TNIK pArg180* (03231) cell line also shows an increase in the spike frequency and number of spikes per second with no changes in the duration of bursts or the number of spikes within bursts. These results confirmed the observed phenotypes with the TNIK p.Arg180* (Patient) cell line (Figures 1H, 2F), suggesting that they are independent of the genetic background used.

### Generation of the TNIK K54R kinase dead mutation iPSC line [TNIK K54R (KD; 03231)]

**Figure 3:**
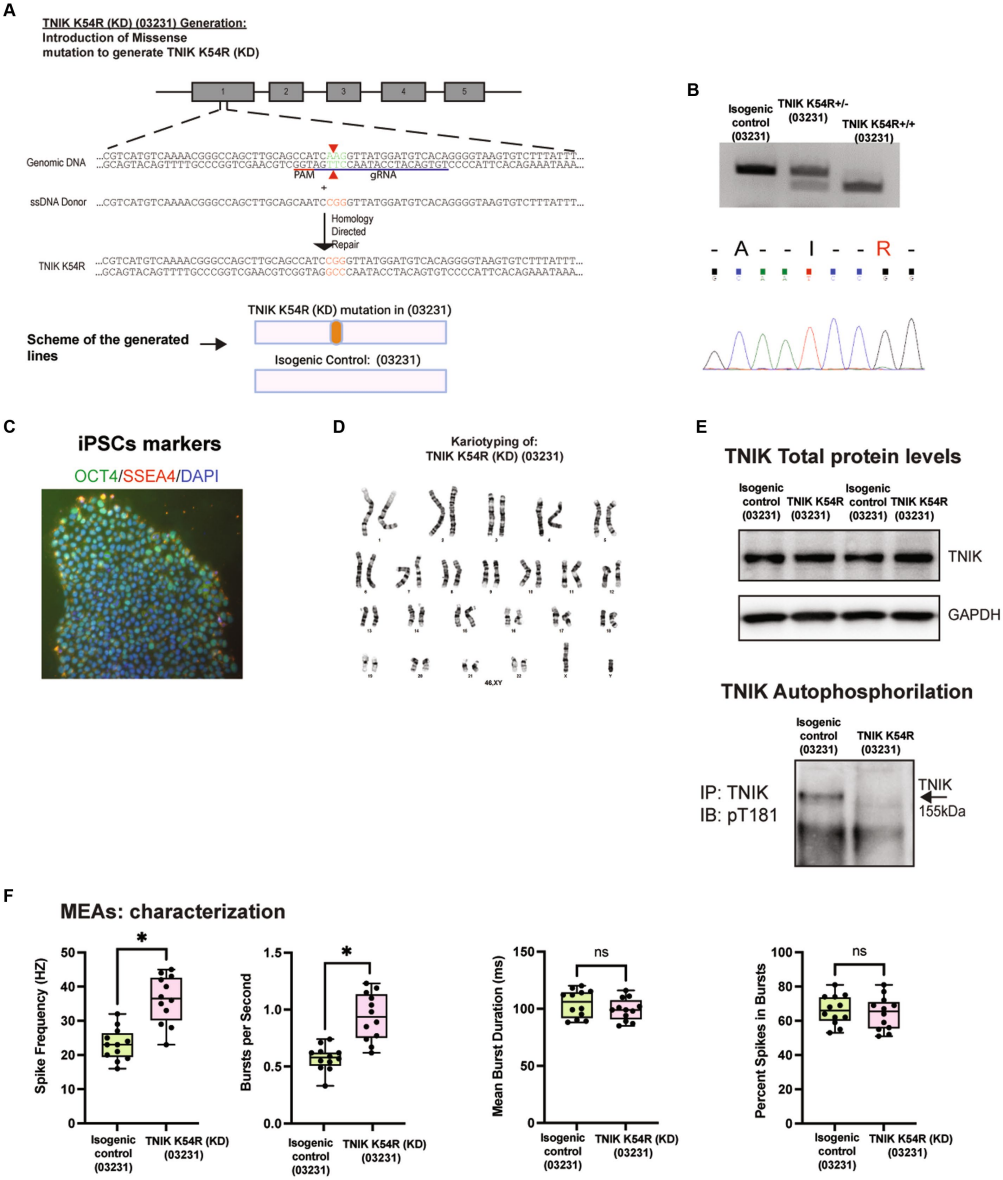
Generation and characterization of TNIK K54R kinase dead (KD; 03231) cell line. **(A)** Diagram illustrating the generation of TNIK K54R (KD) cell line by CRISPR/Cas9 genome engineering in WT 03231 iPSCs, including guide RNA, ssDNA donor, and HDR (Top Panel). Schematic representation of the cell lines generated (Bottom Panel). **(B)** Agarose gels showing polymerase chain reaction followed by restriction enzyme digest screening for TNIK K54R (KD; 03231; Top panel). Sanger sequencing confirmation of cell lines homozygous for TNIK K54R (KD; 03231; Bottom panel). **(C)** Expression of pluripotent stem cell markers OCT4 and SSE4 in TNIK K54R (KD; 03231) iPSCs. **(D)** Karyotyping of TNIK K54R (KD; 03231) cell line shows no gross abnormalities. **(E)** Top panel: Immunoprecipitation of TNIK followed by immunoblot using an antibody directed against TNIK shows normal expression of TNIK protein in TNIK K54R (KD; 03231) cell line compared to its isogenic control. Bottom panel shows immunoprecipitation of TNIK followed by immunoblot using an antibody against TNIK autophosphorylation site (T181). Representative WB shows that TNIK K54R (KD; 03231) has no protein kinase activity in hiPSC-derived neurons. **(F)** Recording of the electrical activity of iPSC-derived neurons using multi-electrode arrays (MEAs): TNIK K54R (KD; 03231) cell line shows the same phenotype as TNIK p.Arg180* (Patient) with increased spikes frequency and number of bursts per second. Each data point represents a recording well. Detection thresholds were set to a fixed level of −25 μV. *p < 0.001.

TNIK has different protein domains that can be responsible for its function; however, the TNIK pArg180* (Patient) mutation generates a TNIK KO. Therefore, to determine whether the lack of TNIK protein kinase activity is enough to disrupt neuronal activity, we generated a TNIK kinase dead cell line by mutating a conserved lysine residue in the ATP-binding pocket of the kinase domain to arginine (K54R; Fu et al., 1999). We called this new cell line, TNIK K54R kinase dead (KD; 03231). To be able to compare the results obtained with the TNIK p.Arg180* (Patient) cell line, we generated the TNIK KD in the WT 03231 cell line background. Figure 3 shows the successful generation of the TNIK K54R (KD; 03231)cell line, with the confirmed mutation, genotype, and karyotype (Figures 3A–D). Furthermore, we wanted to confirm that the TNIK K54R (KD; 03231) mutation produced a TNIK protein without protein kinase function. To monitor TNIK protein kinase activity, we generated a phosphorylation site-specific antibody against the TNIK autophosphorylation site, threonine 181 (T181). However, this phosphorylation site (and the consensus phosphorylation sequence) is conserved among TNIK family members, MINK1 and MAP4K4; therefore, to determine TNIK T181 phosphorylation, we first immunoprecipitated TNIK from the isogenic control (03231) and the TNIK K54R (KD; 03231) cell lines in iPSC-derived neurons and then immunoblotted with the phosphorylation site-specific T181 antibody. This assay was able to show a complete lack of TNIK autophosphorylation in the TNIK K54R (KD; 03231) cell line, ensuring that TNIK protein kinase domain was not active (Figure 3E). This experiment also suggests that the phosphorylation of TNIK T181 cannot be compensated by other protein kinases.

### TNIK protein kinase activity is necessary for normal neuronal function in immature excitatory neurons

To further characterize the TNIK protein kinase function, we recorded the activity of iPSC-derived neurons in the TNIK K54R (KD; 03231) cell line (Figure 3F) and observed that the lack of TNIK protein kinase activity replicated the results of TNIK knockout (KO) protein, as observed in both TNIK p.Arg180* (Patient) and TNIK pArg180* (03231) cell lines. This shows that a non-functional protein kinase domain in TNIK is sufficient to disrupt neuronal activity in immature excitatory neurons increasing their firing rate, also confirming the results observed are specific of TNIK function in two different cellular backgrounds and with two different TNIK mutations.

### Lack of TNIK protein kinase activity disrupts synaptic and non-synaptic signaling in immature excitatory neurons

**Figure 4:**
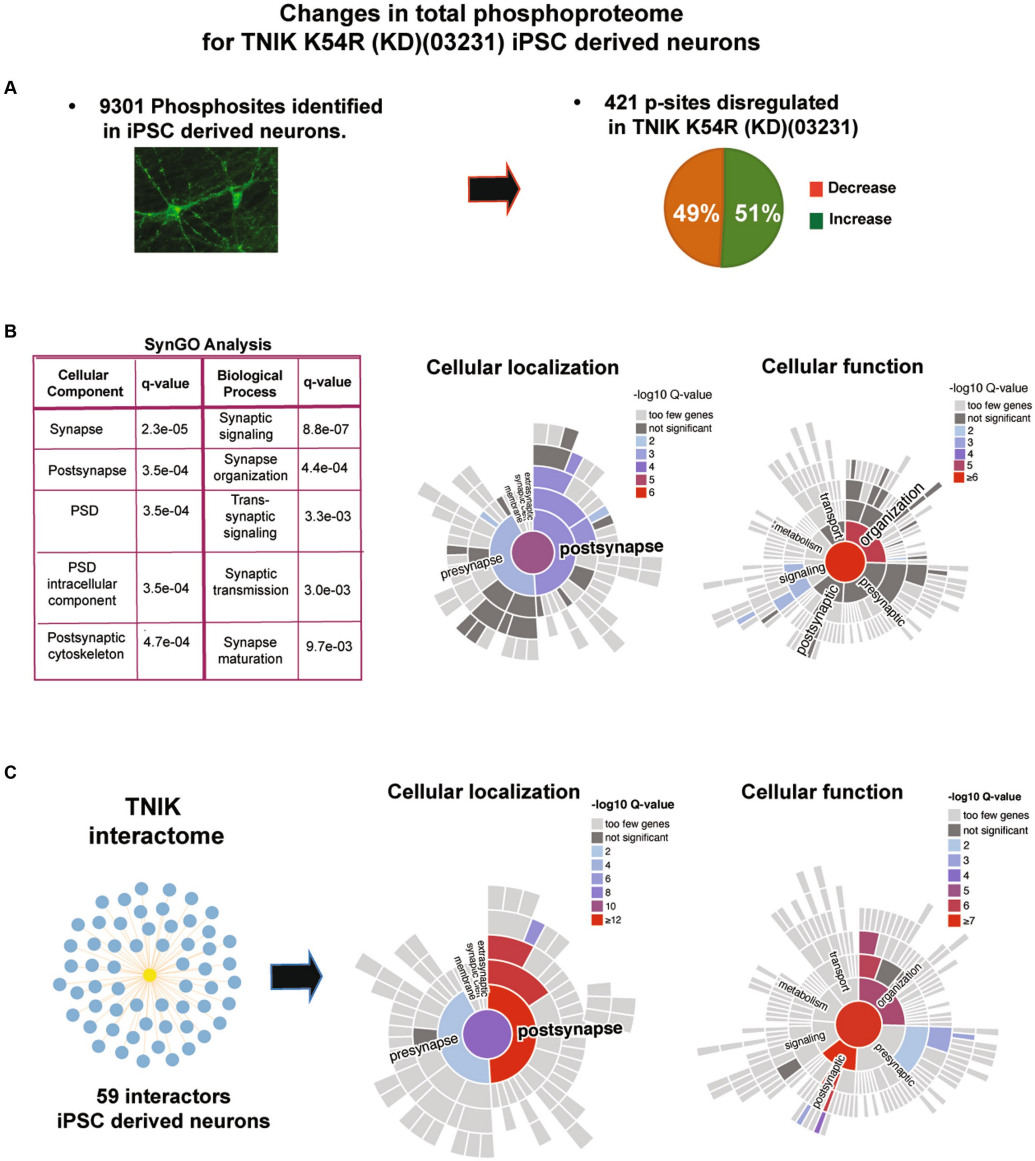
**(A)** Immunofluorescence for iN and results from mass spectrometry analysis of TNIK K54R (KD; 03231) and its isogenic control (03231) iN. Figure shows total numbers of phosphorylation sites identified in iN (9,301 phosphorylation sites) and number of phosphorylation sites showing decrease or increase in protein phosphorylation (dysregulated: 408 phosphorylation sites) in total lysates from TNIK K54R (KD; 03231) iN. **(B)** SynGO analysis of dysregulated p-sites. Analysis shows that the lack of TNIK protein kinase activity dysregulates phosphorylation in proteins localized at the PSD (cellular localization) and proteins associated with the cytoskeletal organization of the PSD (cellular function). **(C)** TNIK interactome isolated from iN. Right panels show SynGO analysis of TNIK interactome with a significant enrichment in PSD proteins and structural components of the PSD. A total neuronal proteome of NGN2 iN was used as background control for SynGO analysis.

To determine the signaling mechanisms disrupted by the lack of TNIK activity in immature neurons, we performed quantitative total protein and protein phosphorylation analysis using HPLC–MS/MS in TNIK K54R (KD; 03231)-induced neurons (iNs) and its correspondent isogenic control (03231). To control that changes in protein phosphorylation are not due to changes in total protein levels, we first determined changes in the proteome of TNIK K54R (KD; 03231) iN (Supplementary Table 1). We then quantitated changes in protein phosphorylation in 9301 phosphorylation sites identified using five replicate assays and identified 408 sites showing dysregulation in TNIK K54R (KD; 03231) iN (Figure 4A; Supplementary Tables 1, 2). TNIK is localized in multiple cellular localizations (Coba et al., 2012; Wilkinson et al., 2019); thus, to analyze what cellular compartments and functions might be interactome in iN. Total proteome and phosphoproteome of TNIK kinase activity, we performed SynGO analysis (Koopmans et al., 2019) of phosphorylation sites upregulated and downregulated in TNIK K54R (KD; 03231) iN. Importantly, to perform SynGO functional and cellular analysis, we used the iN proteome identified in our total proteome assays (6,401 proteins; Supplementary Table 1) and used it as background control of neuronal proteins. Therefore, cellular changes in protein phosphorylation are not affected by the iN proteome. We observed that dysregulated phosphorylation sites were enriched in synaptic proteins, in particular, PSD proteins (Figure 4B). Within the PSD, we found an enrichment in functions related to synaptic organization, transmission, maturation, and signaling processes, suggesting that the main cellular component to be dysregulated by the lack of TNIK protein kinase activity is the postsynaptic density (PSD).

TNIK is able to interact with a variety of proteins in mouse neurons and non-neuronal cells (Camargo et al., 2008; Coba et al., 2012; Li et al., 2016; Wilkinson et al., 2019); however, TNIK protein interactome in iN is not known. In particular, it is not known whether TNIK can also interact with PSD proteins and to what extent the TNIK interactome in iN recapitulates TNIK protein–protein interactions (PPIs) in adult rodent neurons and PSDs. Therefore, we performed a TNIK immunoisolation using a TNIK KO cell line as negative control and identified its interactome using MS assays. We were able to identify 59 protein interactions including several proteins identified in mature PSDs (Supplementary Table 1). Comparison of the TNIK iN interactome with the TNIK interactome obtained from mature rodent PSDs shows a partial overlap with a 23% identity in TNIK PPIs (Supplementary Table 1). This includes intellectual developmental disorder (IDD)-associated proteins such as DLG1, CNKSR2, KALRN, TRIO, CYFIP2, CAMK2A, and SHANK2 (Supplementary Table 1). Moreover, SynGO analysis of TNIK interactome shows a significant enrichment in structural components of the PSD (Figure 4C). Thus, both TNIK interactome and TNIK K54R (KD; 03231) phosphoproteome indicate a role of TNIK in PSDs of iN.

**Figure 5:**
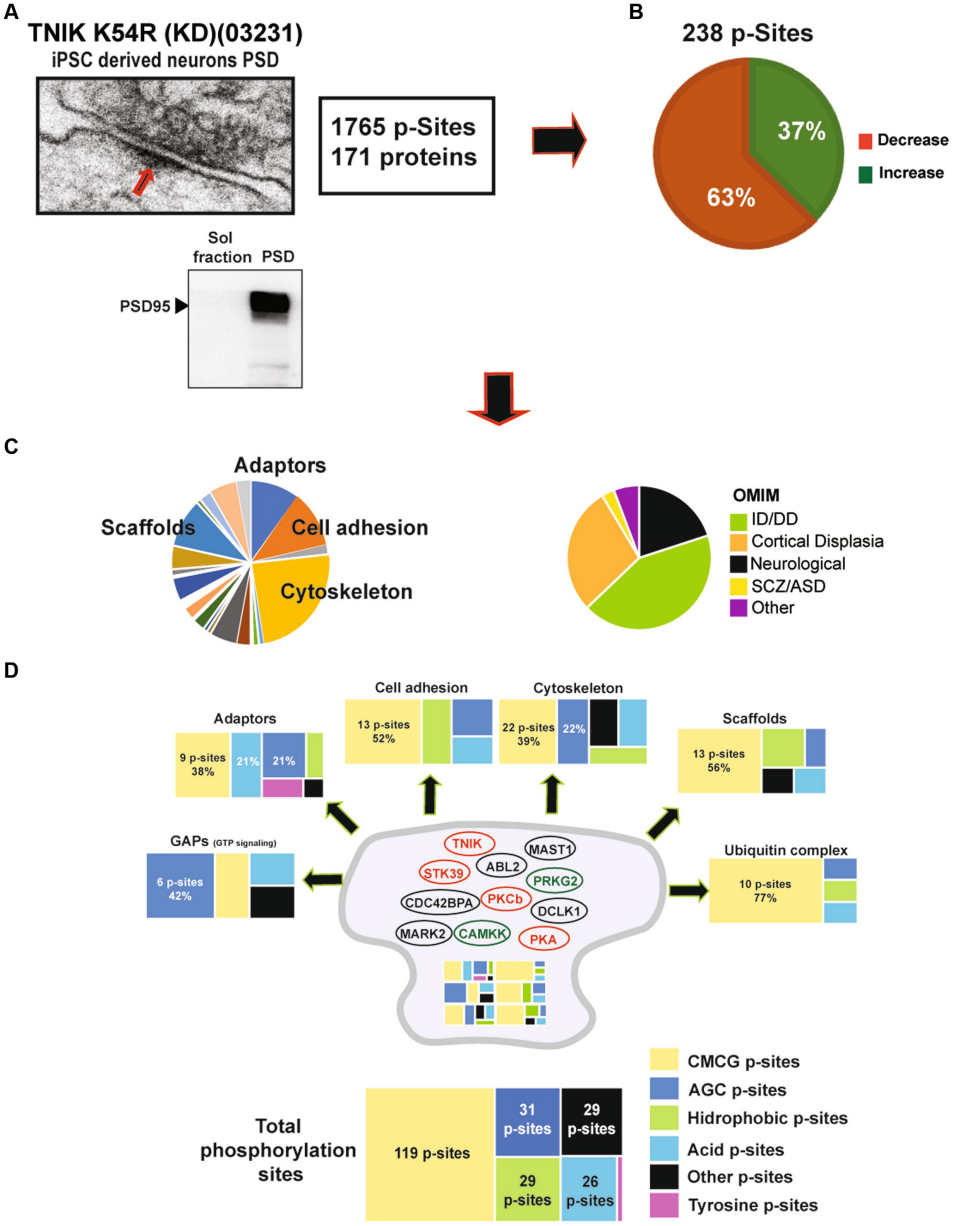
**(A)** Representative electron microscope picture of a synapse including the PSD region from iPSC-derived excitatory neurons. Lower panel: representative WB showing enrichment of the PSD marker PSD95 in PSD fractions when compared to soluble fractions from 21DIV iN. **(B)** Analysis of the phosphoproteome of TNIK K54R (KD; 03231) PSDs. Figure shows a higher percentage of dephosphorylated proteins at the PSD (63%). **(C)** Left pie chart shows protein function categories dysregulated by lack of TNIK protein kinase activity. Chart highlights the top four functional categories: cytoskeletal, adaptors, scaffold, and cell adhesion proteins. Right pie chart shows dysregulated proteins corresponding to genes included in Online Mendelian Inheritance in Man (OMIM) catalog with Mendelian mutations. Chart shows increased numbers of intellectual disability (ID), developmental delay (DD), and cortical dysplasia mutations in components of the PSD from TNIK K54R (KD; 03231) neurons. **(D)** Distribution of protein kinase families predicted to phosphorylate the total number of proteins (bottom) and each functional category (individual panels). Color code shows a large dysregulation of CMCG family of protein kinases across different functional categories together with individual patterns for each functional category. Panels show number of dysregulated phosphorylation sites and individual percentages for most abundant categories. Cartoon of the postsynaptic site shows protein kinases dysregulated in TNIK K54R (KD; 03231) PSDs. Color code shows in red protein kinases with decreased activity and in green protein kinases with increased activity. Protein kinases with dysregulated phosphorylation sites of unknown function are shown in black.

We observed that the lack of TNIK protein kinase activity dysregulates components of the PSD usually reported in mature synapses. Therefore, we decided to isolate PSD fractions (Figure 5A) from TNIK K54R (KD; 03231) iN and perform phosphoproteome analysis by HPLC–MS/MS to address specific changes in the PSD of these iN neurons. We were able to identify 1765 phosphorylation sites in PSD-iN proteins, with a total of 238 phosphorylation sites dysregulated in TNIK K54R (KD; 03231) iN (Figure 5B; Supplementary Table 1). While in the total TNIK K54R (KD; 03231) iN phosphoproteome we determined equivalent numbers of phosphorylation sites showing down- and upregulation (Figure 4A; Supplementary Table 1), the PSD fraction of TNIK K54R (KD; 03231) iN showed a 63% of phosphorylation sites with reduced phosphorylation (Figure 5B; Supplementary Table 1). This suggests a differential spatial regulation of the effects of the lack of TNIK protein kinase activity with a larger dephosphorylation component of the PSD.

The main functional families with changes in protein phosphorylation corresponded to cytoskeletal, cell adhesion, adaptor, and scaffold proteins, suggesting that TNIK K54R (KD; 03231) has a major impact on structural components of the PSD. Dysregulated proteins mapped into different Online Mendelian Inheritance in Man (OMIM) disease categories with the largest number of proteins being causative for developmental delay/intellectual disability (43%) and cortical dysplasia (28%; Figure 5C), suggesting a functional role in synapse development.

**Figure 6:**
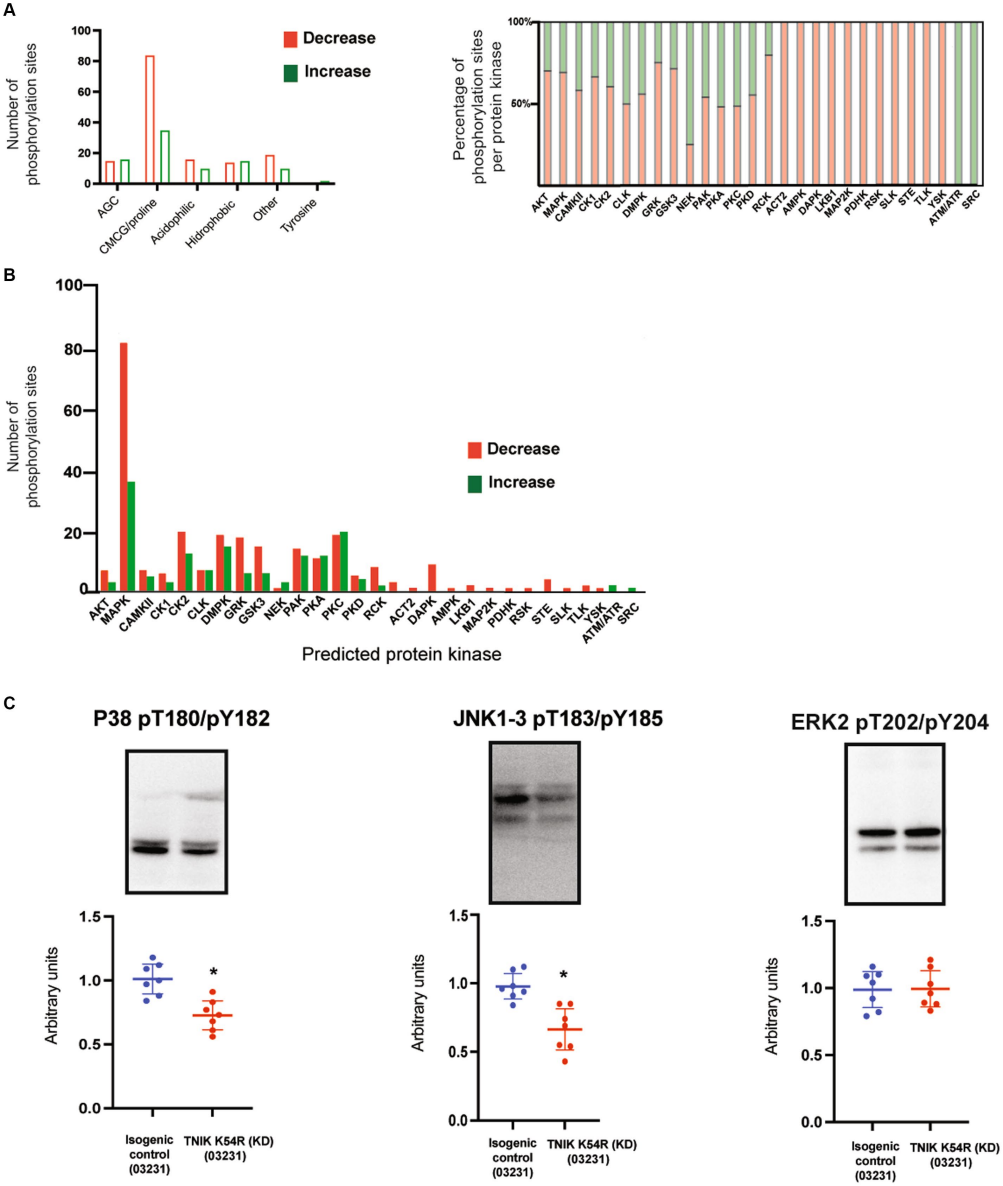
**(A)** Bar graph shows number of phosphorylation sites upregulated (green) and downregulated (red) in iN PSDs. Graph shows kinase families predicted to phosphorylate dysregulated sites. **(B)** Graph shows individual protein kinases predicting to phosphorylate upregulated and downregulated sites at the PSD of TNIK K54R (KD; 03231) neurons, with a larger number of p-sites corresponding to the MAPK family of protein kinases. **(C)** Representative WBs show decreased phosphorylation levels of the P38 family of protein kinases together with JNK1-3 and normal phosphorylation of ERK2 at the PSD of TNIK K54R (KD; 03231) neurons, suggesting a role in the decrease of phosphorylation sites corresponding to MAPK/CMCG kinases. Samples were processed in at least seven replicates per genotype corresponding to two different differentiations. Unpaired *t*-test *p* < 0.001.

We previously reported a functional correlation between protein kinases families and protein functions in mature rodent PSDs (Coba et al., 2009; Li et al., 2016). Thus, we analyzed what protein kinase families were predicted to phosphorylate dysregulated sites within different functional classes of proteins (Supplementary Table 1). We found that most of the dysregulated sites corresponded to protein kinases within the CMCG family (named after **C**DK, **M**APK, **G**SK3, and **C**LK group of kinases). These phospho-sites (p-sites) corresponded to the major structural components of the PSD, including adaptors, scaffolds, cell adhesion, and cytoskeletal proteins (Figure 5D; Supplementary Table 1). Many protein kinases have phosphorylation sites that can be correlated to their own protein kinase activity (activation sites, autophosphorylation sites). Therefore, we examined whether TNIK K54R (KD; 03231) was able to affect protein kinases regulatory sites from the iN PSD kinome (Li et al., 2016). We found that TNIK K54R (KD; 03231) also dysregulated phosphorylation and kinase activity of other protein kinases including STK39, PKCb, PKA, CAMKK, PRKG2 CDC42BPA, ABL2, MAST1, MARK2, and DCLK1 (Figure 5D; Supplementary Table 1). This result is in accordance with the variety of sites other than CMCG that are dysregulated in TNIK iN, among different classes of proteins (Figure 5D; Supplementary Table 1). We then analyzed whether there was a differential effect between downregulated and upregulated phosphorylation sites within functional classes of proteins and with predicted substrate–protein kinase pairs. We found that while most of the kinase families have a similar ratio of upregulated and downregulated phosphorylation sites, the proline directed kinases from the CMCG family have more than two times increase in the ratio of downregulated phosphorylation sites, with more than 80 p-sites showing a decrease in phosphorylation in the PSD fraction of TNIK K54R (KD; 03231) iN (Figure 6A; Supplementary Table 1). We then used NetworKIN to predict protein kinases for each individual phosphorylation site (Linding et al., 2008). As observed for the CMCG family, we found that most of the downregulated phosphorylation sites corresponded to MAPK p-sites (Figure 6B), suggesting a differential effect of TNIK protein kinase activity in specific components of the MAPK family of protein kinases in PSDs.

### TNIK protein kinase activity modulates MAPK signaling in immature PSDs

To confirm the dysregulation of the MAPK family of protein kinases in the PSD of TNIK K54R (KD; 03231) iN, we determined protein phosphorylation of the activation sites for ERK1/2, P38, and JNK1-3 and measured the phosphorylation ratios TNIK K54R (KD; 03231)/TNIK-WT for each protein kinase activation site. Western blot analysis of PSD fractions shows that the lack of TNIK protein kinase activity has no effect in ERK2 phosphorylation and decreases in phosphorylation in the activation sites of P38 and JNK1-3 (Figure 6C), suggesting an impairment in their capacity to phosphorylate proline directed sites at the PSD of immature neurons.

## Discussion

Proteins localized at the PSD of mature excitatory synapses have gained major attention as being associated with neurodevelopmental disorders (Platzer and Lemke, 1993; Radyushkin et al., 2009; Kumar, 2010; Arons et al., 2012; Houge et al., 2012; Coba, 2019; Vlaskamp et al., 2019). On many occasions, highly penetrant truncation mutations are causative of developmental delay or IDD (Li et al., 2017; Kaizuka and Takumi, 2018; Sorokina et al., 2021), and single-nucleotide variants (SNVs) or variants of uncertain significance (VUS) described in complex brain disorders including SCZ, ASD, OCD, and ADHD. Mutations in these set of proteins are usually described as “synaptopathies.” While these proteins are described to be associated in large protein interaction networks, these studies focus on mature rodent models and it is not known whether their protein interaction partners are conserved in developmental synapses, or whether they disrupt the same signaling mechanisms in immature PSDs.

The Traf and Nck interacting kinase (TNIK) is a typical example of this class of PSD proteins. Truncations in TNIK protein are found to be causative of IDD, and while several SNVs and VUS have been described to be associated with SCZ, ASD and ADHD, none of them are statistical significant. However, TNIK interactomes are enriched in risk factors for complex brain disorders. These characteristics are shared with many members of the core signaling machinery of the PSD.

Here, we show that mutations in TNIK disrupt neuronal activity and protein phosphorylation in several PSD proteins considered causative or risk factors for NDD that are also components of the TNIK interactome in iN. Total proteome and phosphoproteome analysis indicate a major dysregulation of structural components of the PSD. Our phosphoproteome analysis of PSD fractions reveals that lack of TNIK protein kinase activity affects immature PSDs by targeting principally their structural components, in particular protein scaffolds, adaptors, cell adhesion, and cytoskeletal molecules. A number of these PSD proteins are also core components of mature rodent synapses such as SHANK2, SHANK3, DLGAP4, and HOMER3. We found that a decrease in phosphorylation in these components is correlated with impaired MAPK activity, specifically in P38 and JNK families of protein kinases. We show that this decrease in protein phosphorylation occurs principally in cytoskeletal proteins, suggesting that the altered synaptic activity in immature neurons might be due to dysregulation of the PSD molecular structure. Interestingly, these pathways seem not to be dysregulated in mature synapses of TNIK^−/−^ mice. However, they are described to be modulated by TNIK activity in non-neuronal cells (Fu et al., 1999). Moreover, a number of members of the STE family of protein kinases like TNIK have also been reported to regulate MAPK activity (Dan et al., 2001), and our results support these roles also in iPSC-derived neurons. In addition, contrary to previous findings in TNIK mature rodent synapses, we did not observe changes in protein phosphorylation or protein content for neurotransmitter receptors of upstream components of the PSD scaffold machinery (Hussain et al., 2010; Wang et al., 2010; Coba et al., 2012; Li et al., 2017). Here, TNIK seems to dysregulate middle and lower layers of the scaffold machinery of the PSD and its cytoskeletal component, and TNIK protein kinase activity might be used to regulate their structural components. Within this signaling structure, we observed 56 proteins being dysregulated that are usually found in the adult rodent PSD PIN, and several of them are also associated with a variety of NDD and are part of the TNIK interactome. Therefore, mutations in these components might also dysregulate shared components of these signaling networks. Our data suggest that PPI and overall signaling networks in immature neurons only partially overlap to classical models of mature PSD signaling. We previously reported similar differences in TNIK interactomes from mature and embryonic day 14 mouse cortex (Li et al., 2017), suggesting that the observed differences are likely to represent changes related to developmental stage rather to differences in species or *in vitro*/*in vivo* methods. Therefore, future studies addressing the role of NDD risk factors at the PSD will need to consider their signaling mechanisms not only in mature synapses but also at early stages of neuronal development. This might help to define phenotypes in accordance with protein function at different stages of brain development.

## Methods

### Postsynaptic density preparation

Postsynaptic density preparations were performed as previously described (Coba et al., 2012). In brief, 8-week-old iN neurons were homogenized in sucrose buffer (0.32 M sucrose, 10 mM HEPES buffer pH 7.4), with 2 mM EDTA, 30 mM NaF, 20 mM β-glycerol phosphate, 5 mM sodium orthovanadate, and Roche cOmplete Protease Inhibitor Cocktail, and centrifuged at 500 *g* for 6 min. Supernatant was collected and then spun at 10,000 g for 10 min. The resulting pellet was solubilized in triton buffer (50 mM HEPES; pH 7.4), 2 mM EDTA, 50 mM NaF, 20 mM β-glycerol phosphate, 5 mM sodium orthovanadate, Roche cOmplete, and 1% Triton X-100. The resulting pellet was collected and solubilized in DOC buffer [50 mM Tris (pH 9), 30 mM NaF, 5 mM sodium orthovanadate, 20 mM β-glycerol phosphate, 20 μM ZnCl_2_, Roche cOmplete, and 1% sodium deoxycholate] and served as the PSD fraction.

### Immunoprecipitation

TNIK immunoprecipitation (IP) in induced neurons (iNs): iPSC-derived iNs in triplicate samples cultured in geltrex coated 10 cm tissue culture dishes were harvested via accutase treatment and lysed in DOC cell lysis buffer using mechanical homogenization followed by incubation at 4 degrees Celsius while rotating for 40 min. The cell lysate was then centrifuged at 35,000 RPM for 30 min at 4 degrees Celsius, and the protein concentration of the supernatant was subsequently determined using the BCA assay (Thermo Fisher Scientific, Waltham, MA). iN 2 mg of total protein was mixed with 50 ul of Anti-TNIK antibody (Bethyl Laboratories, catalog #A302-695A), incubated at 4 degrees Celsius overnight with gentle rotation, and then incubated 2 h at 4C with Magnetic Beads (Sigma). Beads were washed three times in IP wash buffer (25 mM Tris pH 7.4, 150 mM NaCl, 1 mM EDTA, 1% Triton), and then, magnetic beads were boiled at 95 degrees Celsius in 2X LDS buffer, followed by in-gel digestion and MS analysis as described in Li et al. (2017). Triplicate samples from 03231 iN were used for analysis and TNIK KO iN used as controls. Positive interactions were considered by two peptides being present in triplicate samples and absent in TNIK KO control. Primary antibodies used for immunoprecipitation in this study included TNIK (Bethyl Laboratories, catalog #A302-695A, used at 1μg/μl).

### Mass spectrometry and data processing

TNIK K54R (KD; 03231) iPSCs and isogenic control were differentiated to immature cortical neurons for 2 weeks. Pellets were collected and flash-frozen in liquid nitrogen. Samples were processed in at least four replicates from two independent differentiations per each genotype.

Cell pellets from five replicates per genotype were lysed in urea lysis buffer (8 M Urea, 50 mM HEPES pH 8.2, 10 mM glycerol-2-phosphate, 50 mM NaF, 5 mM sodium pyrophosphate, 1 mM EDTA, 1 mM sodium vanadate, 1 mM DTT, 500 nM okadaic acid, complete protease inhibitor cocktail, phosphatase inhibitor cocktail III). After protein concentration was measured using Pierce BCA assay, 100 μg of each sample was used further for multiplexed quantitative proteomics giving a total of 1 mg protein. Each sample was reduced with 10 mM TCEP for 1 h at 55 degrees and alkylated with 20 mM IAA for 30 min in the dark at room temperature. This was followed by quenching with DTT in 20 mM final concentration. Urea samples were then diluted with 50 mM HEPES pH 8.5 before being digested with LysC (Lysyl endopeptidase, 125–05061, FUJIFILM Wako Chemicals) and trypsin (MS grade, 90,058, Thermo Fisher Scientific) at 37 degrees shaking overnight. Each sample was then tandem mass tag (TMT) labeled for an hour at room temperature using a TMT10plex Isobaric Label Reagent Set (0.8 mg per tag, 90,110, Thermo Fisher Scientific, LOT TJ268160) and following manufacturer’s instructions. A small aliquot of each sample was collected for a labeling efficiency and mixing accuracy quality checks (QCs) by liquid chromatography tandem mass spec (LC–MS/MS) using Orbitrap Fusion Lumos Mass Spectrometer and a 60-min HCD MS2 fragmentation method. The rest of the sample was stored at −80 degrees until QC results. Labeling efficiency of higher than 99% was obtained for each reaction and a mixing accuracy with lower than 1.5x difference between samples with lowest and highest summed intensity. Samples were defrosted, quenched with hydroxylamine for 15 min at room temperature, and pooled together. Combined mixture was partially vacuum-dried and acidified to pH 2.0 followed by sample clean-up using C_18_ Sep-Pak Vac 1 cc, 50 mg bed volume (Waters) with gravity flow. Final mixing check was performed by LC–MS/MS with a 240 min HCD MS2 fragmentation method. The peptide mixture was then subjected to high-select sequential enrichment of metal oxide affinity chromatography (SMOAC) to capture phospho-peptides. It was first passed through a high-select TiO_2_ phosphoenrichment column (Thermo Scientific, A32993) following manufacturer protocol. Flow-through and wash fractions were combined, dried, and subsequently used for Fe-NTA phosphoenrichment (Thermo Scientific, A32992). One-tenth of the combined flow-through and wash fractions from this enrichment was used for total proteome analysis. The eluates from SMOAC were freeze-dried, solubilized, and pooled together. Total proteome sample was further subjected to high pH reversed phase fractionation (Thermo Scientific, 84,868), dried, and resolubilized in 0.1% TFA prior to LC–MS/MS. Peptides were separated on a 50 cm, 75 μm I.D. PepMap column over a 2-h gradient and eluted directly into the mass spectrometer (Orbitrap Fusion Lumos) and fragmented using HCD MS2 and SPS MS3. Xcalibur software was used to control the data acquisition. The instrument was run in data-dependent acquisition mode with the most abundant peptides selected for MS/MS by HCD fragmentation.

Raw data were processed using MaxQuant v1.6.2.10 and searched against Uniprot *Homo sapiens* complete proteome canonical sequences from May 2018. Processed data were then analyzed in Perseus, filtered for reverse database hits, log2 transformed, and compared using Welch’s *t*-test with a threshold *p*-value <0.05.

PSD fractions and phospho-peptide enrichment were prepared following the same protocol used for mouse brain tissue as described before (Li et al., 2016). In brief, iPSC-derived neurons were cultured for 8 weeks; we isolated PSD fractions from four replicates of TNIK K54R (KD; 03231) cell line and their correspondent isogenic control and, following enrichment of phospho-peptides with titanium dioxide (TiO_2_) chromatography, samples were analyzed using LC–MS/MS. Samples were reconstituted in LC buffer A (0.1% formic acid in water), randomized, and then injected onto an EASY-nLC 1,200 ultra-high-performance liquid chromatography coupled to a Q Exactive Plus quadrupole-Orbitrap mass spectrometer (Thermo Fisher Scientific). Peptides were separated by a reverse phase analytical column (PepMap RSLC C18, 2 μm, 100 Å, 75 μm × 25 cm). Flow rate was set to 300 nL/min at a gradient from 3% LC buffer B (0.1% formic acid, 80% acetonitrile) to 38% LC buffer B in 110 min, followed by a 10-min washing step to 85% LC buffer B. The maximum pressure was set to 1,180 bar, and column temperature was maintained at 50°C. Peptides separated by the column were ionized at 2.4 kV in positive ion mode. MS1 survey scans were acquired at the resolution of 70,000 from 350 to 1,800 m/z, with a maximum injection time of 100 ms and AGC target of 1e6. MS/MS fragmentation of the 14 most abundant ions was analyzed at a resolution of 17,500, AGC target 5e4, maximum injection time 65 ms, and normalized collision energy of 26. Dynamic exclusion was set to 30 s, and ions with charge +1, +7, and > +7 were excluded. MS/MS fragmentation spectra were searched with Proteome Discoverer SEQUEST (version 2.2, Thermo Scientific) against *in silico* tryptic digested Uniprot all-reviewed *Homo sapiens* database (release June 2017, 42,140 entries). The maximum missed cleavages were set to two. Dynamic modifications were set to phosphorylation on serine, threonine, or tyrosine (+79.966 Da), oxidation on methionine (+15.995 Da), and acetylation on protein N-terminus (+42.011 Da). Carbamidomethylation on cysteine (+57.021 Da) was set as a fixed modification. The maximum parental mass error was set to 10 ppm, and the MS/MS mass tolerance was set to 0.02 Da. The false discovery threshold was set strictly to 0.01 using the Percolator Node. Individual phospho-site localization probabilities were determined by the ptmRS node, and phospho-sites with <0.75 localization probability were removed. The relative abundance of phospho-peptides was calculated by integration of the area under the curve of the MS1 peaks using the Minora LFQ node in Proteome Discoverer. No data imputation was performed for missing values. Phospho-peptides were filtered so that each condition had at least two quantified values. Phospho-peptide intensities were then normalized by log_2_-transformation and sample median subtraction. Only phosphorylation sites with proteins with no changes in the same direction in total protein levels were used for further analysis.

### Cell culture and neural differentiation

#### iPSC culture

The control 03231 iPSC line was generated from a lymphoblastoid cell line derived from a healthy 56-year-old male (NINDS repository, ND03231) as previously described (Pepper et al., 2016). iPSCs were cultured on Geltrex (Thermo Fisher Scientific) coated plates in mTeSR-1 medium (Stemcell Technologies, Vancouver, Canada) at 37 degrees Celsius and 5% CO_2_. Karyotyping was carried out by the Center for Personalized Medicine at the Children’s Hospital of Los Angeles.

### Induced neuron differentiation

Induced neurons (iNs) were differentiated directly from iPSCs using a modified protocol derived from Zhang et al. (2013). When confluent, iPSCs were split via Accutase and seeded into Geltrex coated 6-well plates at a density of 3.0 × 10^5^ cells per well in mTeSR supplemented with 10 μM rock inhibitor Y-27632. The next day, the media were changed to mTeSR supplemented with 4 μg/mL polybrene, and iPSCs were infected with hNGN2 (Addgene plasmid # 79049) and rtTA (Addgene plasmid # 19780) lentiviruses at a concentration to obtain >90% infection efficiency. Media were changed daily until iPSCs were ready for passaging. iPSCs were then passaged directly into neural induction media (DMEM/F12 basal media), 1X N2 Supplement, 1X NEAA, 10 ng/mL BDNF (Shenandoah Biotechnology, Warwick, PA), 10 ng/mL NT-3 (Shenandoah Biotechnology), and 1 μg/mL Doxycycline (Enzo Life Sciences, Farmingdale, NY) supplemented with 10 μM rock inhibitor Y-27632. Cells were seeded at a density of 2.0 × 10 (Ting et al., 2012) cells per well in 6-well plates. The next day, media were changed to neural induction media supplemented with 0.7 μg/mL puromycin. 48 h later, media were changed to B27 media (Neurobasal, 1X B27 Supplement, 1X Glutamax, 10 ng/mL BDNF, 10 ng/mL NT-3, and 1 μg/mL doxycycline). 48 h later, media were changed to B27 media supplemented with 2 μM Ara-C (MilliporeSigma, Burlington, MA). Half media changes were carried out every other day until neurons were harvested at 3 weeks post-initiation of differentiation.

### Cloning

For gene editing, the guide RNA sequences were designed using the CRISPR design tool.^1^ Oligonucleotides were ordered from Integrated DNA Technologies (San Diego, CA) and cloned into pSpCas9(BB)-2A-Puro (PX459) V2.0 (Addgene plasmid # 62988) as described by Ran et al. (2013). Insertion of the guide sequence was confirmed via Sanger sequencing.

### Gene targeting

When confluent, iPSCs were harvested via accutase treatment, and 1.5 × 10 (Trubetskoy et al., 2022) cells were nucleofected with 6 μg PX459 (Addgene plasmid # 62988) with a TNIK-specific sgRNA cloned into the BbsI restriction enzyme sites and 1 μL of a 100 μM custom HDR template ordered as Ultramer DNA oligos [single-stranded oligonucleotide (ssODN)] from Integrated DNA Technologies using the Amaxa NHDF nucleofector kit (Lonza, Basel, Switzerland) with program B-016. Nucleofected cells were split in equal numbers between 2 and 4 wells of a 6-well plate in mTeSR-1 media supplemented with rock inhibitor. A 48-h selection period was then started with 0.5 μg/mL puromycin. Cells were fed daily until colonies became apparent. Individual colonies were transferred into 24-well plates and allowed to grow until genotyping was subsequently carried out.

### Lentiviral production

HEK293 cells cultured on 0.1% gelatin coated tissue culture dishes were transfected using polyethylenimine (Polysciences Inc., Warrington PA) at approximately 90% confluency with viral vectors containing the gene of interest and lentiviral packing plasmids (pPAX2 and VSVG). The medium was changed 24 h after transfection, and then medium containing lentiviral particles was harvested at 48 and 72 h after transfection. Medium containing lentiviral particles was filtered using 0.45 μM filters and then concentrated using Lenti-X concentrator (Clontech, Mountain View, CA).

### Western blotting

Samples were processed in at least four replicates per each genotype and loaded on 4–12% Bis-Tris gels (NuPAGE Novex, Thermo Fisher Scientific) and separated at 135 V for 1.5 h. Proteins were then transferred to a PVDF membrane using a Bio-Rad Trans-Blot Turbo Transfer System (Bio-Rad, Hercules, CA). Membranes were blocked for 1 h at room temperature with 5% bovine serum albumin (BSA) in 0.05% TBS-Tween (TBST) and then incubated with primary antibody at 1:1000 dilution overnight at 4 degrees Celsius. Primary antibodies used here include TNIK (Thermo Fisher Scientific, catalog #PA1-20639), TNIK (Bethyl Laboratories, catalog #A302-695A), Phospho-TNIK – Thr181 (custom antibody), P38 pT180/pY182 (Cell Signaling catalog #4511), JNK1-3 pT183/pY185 (Cell Signaling catalog # 8328), and ERK2 pT202/pY204 (Cell Signaling catalog #4370). Membranes were washed with 0.05% TBST four times, 10 min each, and then incubated with secondary antibodies for 1 h at room temperature. Membranes were washed with 0.05% TBST four times, 5 min each, and imaged using a 4000MM Pro Image Station (Carestream, Rochester, NY).

### Multi-electrode array recordings

iPSC lines were cultured on Geltrex (Thermo Fisher Scientific, Waltham, MA) coated plates with mTeSR-1 plus medium (Stemcell Technologies, Vancouver, Canada) at 37 degrees Celsius and 5% CO2. iPSCs were gently washed with DMEM (Lonza, Basel Switzerland) and harvested via accutase treatment. The cells were then centrifuged at 1000 RPM for 5 min, resuspended in mTesR plus medium with 10uM rock inhibitor Y-27632 (Sigma-Aldrich, St. Louis, MO), and seeded at a density of 3×10^5 cells per well into 6-well plates (Genesee Scientific, San Diego, CA). After 24 h, the cells were infected with hNGN2 and rtTA lentivirus with polybrene (1:2000; Sigma-Aldrich, St. Louis, MO) in mTesR plus medium, as previously described (Zhang et al., 2013). The next day, a 48-h selection period was started with 0.5 ug/ml puromycin (Thermo Fisher Scientific, Waltham, MA). After the puromycin selection period, the cells are fed every other day with complete mTesR medium until they reach 80–90% confluency. The confluent cells are gently washed with DMEM, harvested via accutase treatment, and centrifuged at 1000 RPM for 5 min. The pellet is resuspended in N2 media (DMEMF/12, 1X N2 Supplement, 1X penicillin–streptomycin, 1X NEAA Supplement, 10 ng/mL BDNF, 10 ng/mL NT3, 1ug/ml doxycycline) and re-plated at a density of 1×10^6 cells per 10 cm^2 dish (VWR, Radnor, PA). The next day, a 48-h selected period was started with 0.5ug/ml puromycin. After the puromycin selection period, the cells are harvested via accutase treatment and centrifuged at 1800 RPM for 7 min. The cell pellet was resuspended in Neurobasal/B27 medium supplemented with 10uM rock inhibitor, and dissociated cells were seeded in the center of poly-L-ornithine/laminin-coated MEAs (60MEA200/30-Ti, Multi-Channel Systems, Reutlingen, Germany) at a density of 5×10^4 cells per well. The plated MEAs are transferred to tissue culture incubators maintained humidified at 37°C and 5% CO2/95% air. The following day, murine primary astrocytes (ScienCell Research Laboratories, Carlsbad, CA) are harvested via trypsin treatment and seeded into the MEA wells containing neurons. The next day, the Neurobasal/B27 media are supplemented with 2 uM Ara-C (1-β-D-Arabinofuranosylcytosine; Sigma-Aldrich, St. Louis, MO). Half medium changes are done every other day for 3 weeks. After the 3 weeks, Neurobasal/B27 medium is supplemented with 2.5% fetal bovine serum (Genesee Scientific, San Diego, CA) for astrocyte viability, and half medium changes are done once a week until the end of the culture period.

MEAs consist of 8 by 8 grids of titanium nitride electrodes of 30 μm diameter and interelectrode spacing of 200 μm. Local field potentials (LFPs) were recorded from iPSC-derived neurons after 8 weeks in culture in six-well multi-electrode chips (nine electrodes and one ground per well) using a Multi-Channel Systems MEA-2100 multi-electrode array (MEA) amplifier (ALA Scientific) with built-in heating elements set to 37°C. Samples were processed in at least four replicates from two different differentiations per each genotype. Cells were allowed to acclimate for 5 min after chips were placed into the MEA amplifier. For each condition, recordings were filtered between 1 and 500 Hz, and average LFP frequency per well was determined using the accompanying MC Rack software. Spontaneous spikes detected in the MEAs were analyzed using MC Rack (Multi-Channel Systems), and action potentials were digitized with a 128-channel analog/digital converter card by sampling the 1 ms pre- and 2 ms post-crossing of threshold at a rate of 25 kHz. Cell cultures were analyzed in at least four replicate samples per genotype, using at least two different differentiations. Detection thresholds were set to a fixed level of −25 μV. ANOVA and Fisher’s PLSD post-hoc test were used to statistically analyze the quantitative data.

### Immunofluorescence

Cells were fixed in 4% paraformaldehyde at room temperature for 20 min, washed three times with PBS, and then permeabilized with 0.5% PBS-Tween (PBST) overnight. Cells were then blocked with blocking solution (10% fetal bovine serum in 0.1% PBST) for 1 h at room temperature and then incubated with the primary antibody diluted in blocking buffer overnight at 4 degrees Celsius. Primary antibodies used here include the following: OCT4 (Cell Signaling Technology, Cat. 2,840) 1/400, SSEA4 (Cell Signaling Technology, Cat. 4,755) 1/500, MAP2 (Cell Signaling Technology, Cat. 4,542) 1/400, NESTIN, (BioLegend, Cat. 656,802) 1/500, SOX2 (Cell Signaling Technology, Cat. 3,579) 1/400, PAX6 (BioLegend, Cat. 901,301) 1/300. The next day, the cells were washed three times with 0.1% PBST and then incubated with the secondary antibody for 1 h at room temperature followed by DAPI staining.

### Enrichment analysis

Gene ontology enrichment analysis was carried using SynGO (Koopmans et al., 2019). Only terms that were significant following the Bonferroni correction for multiple comparisons are reported in the text.

### Transmission electron microscopy

After 8 weeks, induced neurons were fixed at room temperature with 4% paraformaldehyde, 2.5% glutaraldehyde, and 7% sucrose in 0.1 M HEPES buffer (pH 7.4) for 3 h and left overnight in a fixative at 4°C. After washing for 3 × 10 min in cold PBS (phosphate-buffered saline, pH 7.2), samples were then fixed for 1 h with 2% osmium tetroxide in PBS, pH 7.2. They were washed 3 × 10 min in PBS and 1 × 10 min in ddH_2_O at RT and stain with 2% uranyl acetate. Sample were then washed three times with ddH_2_O at RT and dehydrated in an ascending ethanol series using solutions of 50, 70, 90, 100, 100, and 100% 10 min each. Samples were then incubated two times in propylene oxide as a transition to infiltration with Eponate 12 (Ted Pella Inc.) starting with 1:1 propylene oxide to Eponate for 1 h, 1:2 for 1 h, and then pure Eponate overnight. The next day, two 1-h incubations in fresh Eponate were made before the samples were placed in molds and polymerized at 60°C for 18 h. Polymerized blocks were trimmed by hand with razor blades and then sectioned on a Leica LC6 ultramicrotome initially at 1 micron to find the area of interest. Then, thin sections (70 nm) were picked up on formvar and carbon-coated copper grids. Grids were stained with lead citrate followed by uranyl acetate. Images were taken on a FEI (Thermo Fisher Scientific) Talos F200C at 80KeV with a Ceta 4 K CMOS camera.

## Data availability statement

The data presented in the study is deposited in the PRIDE repository, accession number PXD035004.

## Ethics statement

The studies involving humans were approved by University of Southern California HS-18-00745-AM003. The studies were conducted in accordance with the local legislation and institutional requirements. The human samples used in this study were acquired from gifted from another research group. Written informed consent for participation was not required from the participants or the participants’ legal guardians/next of kin in accordance with the national legislation and institutional requirements. The animal study was approved by University of Southern California Stem Cell Research Oversight Committee. Protocol #2020–2. Study ID: HS-18-00745-AM003. The study was conducted in accordance with the local legislation and institutional requirements.

## Author contributions

JJ: Data curation, Formal analysis, Methodology, Writing – original draft, Writing – review & editing. BW: Data curation, Formal analysis, Investigation, Methodology, Writing – original draft, Writing – review & editing. IF: Data curation, Investigation, Writing – review & editing. NH: Data curation, Formal analysis, Methodology, Software, Writing – review & editing. SM: Data curation, Formal analysis, Methodology, Software, Writing – review & editing. VC: Data curation, Software, Visualization, Writing – review & editing. HF: Data curation, Methodology, Software, Supervision, Writing – review & editing. FA: Resources, Writing – review & editing. SU: Investigation, Supervision, Data curation, Methodology, Writing – review & editing. NG: Data curation, Methodology, Software, Supervision, Formal analysis, Writing – review & editing. MC: Conceptualization, Data curation, Formal analysis, Funding acquisition, Investigation, Software, Supervision, Writing – review & editing.

## Footnotes

^1^ http://crispr.mit.edu
